# Microevolutionary processes impact macroevolutionary patterns

**DOI:** 10.1186/s12862-018-1236-8

**Published:** 2018-08-10

**Authors:** Jingchun Li, Jen-Pen Huang, Jeet Sukumaran, L. Lacey Knowles

**Affiliations:** 1 Department of Ecology and Evolutionary Biology, University of Colorado Boulder, Boulder, USA; 2 Museum of Natural History, University of Colorado Boulder, Boulder, USA; 3 Integrative Research Center, The Field Museum, Chicago, USA; 4 Museum of Zoology, Department of Ecology and Evolutionary Biology, University of Michigan, Ann Arbor, USA

**Keywords:** Protracted speciation, Speciation duration, Birth-death model, Latitudinal gradient, Microevolution

## Abstract

**Background:**

Macroevolutionary modeling of species diversification plays important roles in inferring large-scale biodiversity patterns. It allows estimation of speciation and extinction rates and statistically testing their relationships with different ecological factors. However, macroevolutionary patterns are ultimately generated by microevolutionary processes acting at population levels, especially when speciation and extinction are considered protracted instead of point events. Neglecting the connection between micro- and macroevolution may hinder our ability to fully understand the underlying mechanisms that drive the observed patterns.

**Results:**

In this simulation study, we used the protracted speciation framework to demonstrate that distinct microevolutionary scenarios can generate very similar biodiversity patterns (e.g., latitudinal diversity gradient). We also showed that current macroevolutionary models may not be able to distinguish these different scenarios.

**Conclusions:**

Given the compounded nature of speciation and extinction rates, one needs to be cautious when inferring causal relationships between ecological factors and macroevolutioanry rates. Future studies that incorporate microevolutionary processes into current modeling approaches are in need.

**Electronic supplementary material:**

The online version of this article (10.1186/s12862-018-1236-8) contains supplementary material, which is available to authorized users.

## Background

Understanding the formation of large-scale biodiversity patterns, such as latitudinal gradient and hyper-diverse lineages, remains a major challenge in ecology and evolutionary biology [1]. A primary objective of this research is to identify and characterize the processes that are responsible for generating differential species diversity among geographical regions or distinct clades [2]. Numerous studies [3–6] have shown that species diversity can be influenced by both extrinsic (e.g., energy supply, environmental stability, climate) and intrinsic (e.g., dispersal ability, adaptive traits) factors. These factors ultimately inform the lineage diversification process through a combination of speciation and extinction events. Therefore, a large body of macroevolutionary studies (both paleontological and neontological) is dedicated to analyzing speciation and extinction patterns and their relationships with various ecological factors [7–11].

Mathematical modeling of speciation and extinction dynamics plays an important role in quantitative inference of macroevolutionary processes, especially when combined with large-scale phylogenetic data [12–14]. The most commonly used framework is the birth-death model and its variations. The model assumes that phylogenetic lineages accumulate with a rate of *λ* - *μ*, where *λ* is the speciation rate and *μ* is the extinction rate [15]. Earlier models presume rates to be constant through time and among lineages, while recently developed models have begun to incorporate rate heterogeneity [13], such as density-dependent [16], trait-dependence [17], or geography-dependence [18] rate shifts within the phylogeny. Empirical rates of speciation, extinction, or rate shifts are estimated to maximize the likelihood of a given phylogeny [19]. These rates can then be compared among clades, or used to statistically test if observed diversity patterns are associated with biological traits, geographical events, or other environmental factors [13, 18, 20–23].

There is no question that the development of macroevolutionary models has enabled the testing of important hypotheses. For example, many historical or ecological factors have been proposed to explain the latitudinal diversity gradient (LDG) of species richness [24, 25], aiming to test the classic hypotheses that the tropics are a cradle (generates diversity, high speciation) or a museum (accumulates diversity, low extinction) of diversity [26]. Paleontological and ecological evidence have shown that the tropics could be both because many higher taxonomical groups preferentially originated from the tropics and remained in the tropics [27]. However, these hypotheses, and perhaps others, cannot be fully tested without joint consideration of both rates of speciation and extinction between the tropics and the higher latitudes. For example, recent model-based studies have shown that the high species richness in the tropics is not a simple result of high speciation (i.e., cradle; [1]). At least for certain taxonomic groups, speciation rates have been found to be higher in temperate zones than in the tropics [28, 29]. These non-intuitive findings have fueled the development of alternative hypotheses for LDG, such as fast turnover rates (high speciation and high extinction) at high latitudes due to environmental harshness, higher ecological opportunity, and extinction by nascent species fusion [1, 30–32]. Because such hypotheses focus on the early stages of speciation, testing them requires a better understanding of microevolutionary dynamics in the diversification process. Yet, these dynamics are largely neglected in current models.

**Fig. 1:**
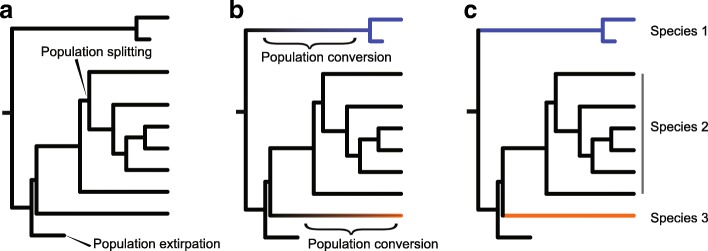
**a** A protracted speciation genealogy where each tip represents a population, and the rate of population splitting (hereafter denoted as *λ’*), conversion (*χ*), and extirpation (*μ’*) all influence the lineage diversification process and ultimately species richness [34]. **b**. Converting events (gradient bars) shown on the genealogy; all descendants after a converting event are considered a new species. **c**. Final species richness after protracted speciation. In this case, three extant species (blue, black and orange) were formed, with species 2 represented by multiple populations

Macroevolutionary patterns are ultimately generated by microevolutionary processes acting at population levels, especially considering that speciation and extinctions are typically protracted instead of point events [33–36]. The process between initial population divergence and formation of a full-fledged species could be complex and is influenced by any number of ecological mechanisms, all of which can contribute to differential rates of lineage diversification [32, 36]. The idea of speciation being compounded with the forming of incipient species and their persistence (ephemeral speciation) is old, tracing back at least to Mayr (1963) [37, 38]. This model was later turned into a protracted speciation framework [36]. In this framework, within-species lineages are considered basic units of diversification. Proliferation of the lineages is subject to three major events: population splitting, population conversion, and population extirpation (Fig. 1). Population splitting represents the initial divergence and reduction of gene flow between different within-species lineages, often resulting from geographical isolation or ecological differentiation; population conversion indicates the formation of a fully reproductively isolated “good” species; population extirpation can be caused by either the death of all members of the within-species lineage or the lineage merging back to its original gene pool [34, 35, 39]. The latter two events also correspond to the “length of speciation duration” and “population persistence” controls of speciation (see [36]).

Application of this protracted species framework, as opposed to traditional birth-death models, have the potential to disentangle the causes underlying differences in species richness among regions. For example, the “high turnover rates” hypothesis [28, 30, 32] predicts that lineages at higher latitude should experience relatively higher rates of population splitting and conversion, as well as higher population extirpation, resulting in overall low species richness compared to the tropics. These different processes can only be distinguished if the protracted speciation model is incorporated into macroevolutionary analyses.

In this study, we demonstrate that population level dynamics can impact macroevolutionary patterns, and that current macroevolutionary models may not discriminate among processes, resulting in difficulties discerning underlying causes of the formation of biodiversity patterns. Specifically, we first provide an example of how contrasting mechanisms can result in a latitudinal gradient in birds by simulating plausible scenarios under a protracted speciation process using empirical data from Weir and Schluter 2007 [28]. We then demonstrate that birth-death based models cannot effectively distinguish between contrasting explanations for observed diversity gradients based on phylogenetic data. We hope these results will promote an awareness of the impact of microevolutionary mechanisms on macroevolution processes and fuel future methodological research to better integrate the two.

## Methods

We conducted two analyses to explore both the variety of processes that might produce species diversity gradients and whether these differences can be detected by traditional macroevolutionary models (i.e., those from a the birth-death analytical framework). For the first set of analyses, we simulated different processes that might generate latitudinal diversity gradients (e.g., differences in opportunities for population divergence, conversion, or population extinction), focusing on the possibility that speciation rates might actually be higher in temperate regions, despite the lower species diversity. In the second set of analyses we tested if these processes that are explicit under a protracted speciation model can be detected by birth-death based macroevolutionary models.

### Latitudinal diversity gradient in birds

Two separate scenarios representing temporal and tropical bird diversification conditions were simulated. The first set of simulations is based on parameters derived from empirical estimations in Weir and Schluter 2007 [28]. In their study, bird speciation rates were estimated as 0.58 in temperate and 0.17 in tropical regions; extinction rates were 0.45 in the temperate zone and 0.04 in the tropics. Based on their estimates of the average sister species divergences times, *t,* of ~ 1 million year for higher latitudes and ~ 3.4 million years for the tropics, we calculated a population conversion rate (*χ*) for each region as 1/2 *t*, or specifically, *χ* = 0.5 and 0.15 for the temperate and tropical regions, respectively. In addition, because speciation rate (*λ*, estimated from Weir and Schluter 2007 [28]) is the product of population splitting (*λ*’, number of diverging populations formed per million year) and conversion (*χ*), we estimated the population splitting rate as *λ/χ*, or specifically, *λ’* = 1.16 and 1.13 for the temperate and tropical regions. Lastly, the population extirpation rate is calculated based on the principle that extirpations of all within-species populations result in the extinction of the species. Specifically, the number of populations per species generated in a given time can be represented by *e*^*λ’ × t*^. The number of populations that remains as intra-specific units are *e*^*λ’ × t*^ × (1 - *χ*) – that is, those that do not convert into new species. The species extinction rate (*μ*) is then the population extirpation rate (*μ’*) to the power of *e*^*λ’ × t*^ × (1 - *χ*). When we consider extinction rate as the rate per million years, *t* can be simplified to 1. Based on known values of *λ’*, *χ* and *μ*, we calculated the *μ’* values as 0.6 and 0.3 for the temperate and tropical regions, respectively.

The second set of simulations is based on a hypothetical scenario where population conversion rates were the same for temperate and tropical regions. This simulation allowed us to explore whether the observed bird species diversity gradient could be generated without invoking differences in rates of reproductive isolation between the regions. The following two parameters were modified for the temperate regions: the population conversion rate was set to the tropical rate (*χ* = 0.15). The population splitting rate was increased to *λ’* = 1.3. All other rates were kept the same as the first set of simulations.

One hundred simulated phylogenies were generated for each scenario using the “pbd_sim” function in the package PBD [39]. The function takes in population splitting and extirpation rates for good and incipient lineages, conversion rate, and simulation time as parameters, and outputs simulated phylogenies. We did not assume any differences in the splitting and extirpation of good and incipient lineages, the same parameter values (*λ’* and *μ’*) were used for both. The simulation times were held constant for 6 million years (i.e., as opposed to keeping the number of tips constant, Fig. 1; see also [40]). Final species richness was summarized across the total phylogenies. For species with more than one population lineage at the end of the simulation (i.e., multiple divergent population lineages that have not yet been converted into new species), one randomly chosen population lineage was retained to represent that species; all other population lineages were pruned from the simulated phylogenetic tree (i.e., output value ‘stree_random’ from the ‘pbd_sim’ function). In addition to summarizing the number of species, we reported cophenetic distances (i.e. approximation of sister species divergence time) between sister species calculated using the R package ‘ape’ [41] for each scenario. The sister taxa were identified using the is.monophyly function in ape. Specifically, if two taxa form a monophyletic group from the phylogeny, they were identified as sister taxa. Welch’s t-test was used to assess if species richness and sister species divergence time differed significantly between the tropical and temperate regions and different scenarios.

### Estimating speciation rates from protracted genealogies

Using the same “pbd_sim” function in the package PBD [39], we produce phylogenies with different protracted speciation parameters to explore whether different processes can generate similar macroevolutionary empirical patterns. That is, if the traditional macroevolutionary interpretations could be similar despite differences in the underlying generative model.

Specifically, we simulated data under 5 different values for each of the three protracted speciation parameters spanning relatively low to high rates of population splitting (*λ’* from 0.5–0.7), population conversion (*χ* from 0.01–0.21), and population extirpation (*μ’* from 0.25–0.45), resulting in a total of 125 parameter combinations. Here, the rate parameters correspond to the rate of each event (i.e., splitting, conversion, and extirpation) occurrence per unit time (e.g., one million year). For example, a population conversion rate of 0.5 would mean that on average, a newly emerged lineage takes 2 million years to convert to a true species. The simulation times were held constant for 15 million years. For each parameter combination, 200 phylogenies were simulated. As described above, only one random sampled population lineage was retained to represent a species when more than one divergent population per species was observed when the simulation ended at 15 million years.

For each simulated phylogeny, birth-death based speciation and extinction rates were estimated using the “bd_ML” function in the R package “DDD” [42]. Mean estimated speciation and extinction rates were calculated across the 200 replicate simulated phylogenies for each of the 125 parameter combinations and plotted as a function of the protracted speciation parameters.

## Results

### Latitudinal diversity gradient in birds

**Fig. 2:**
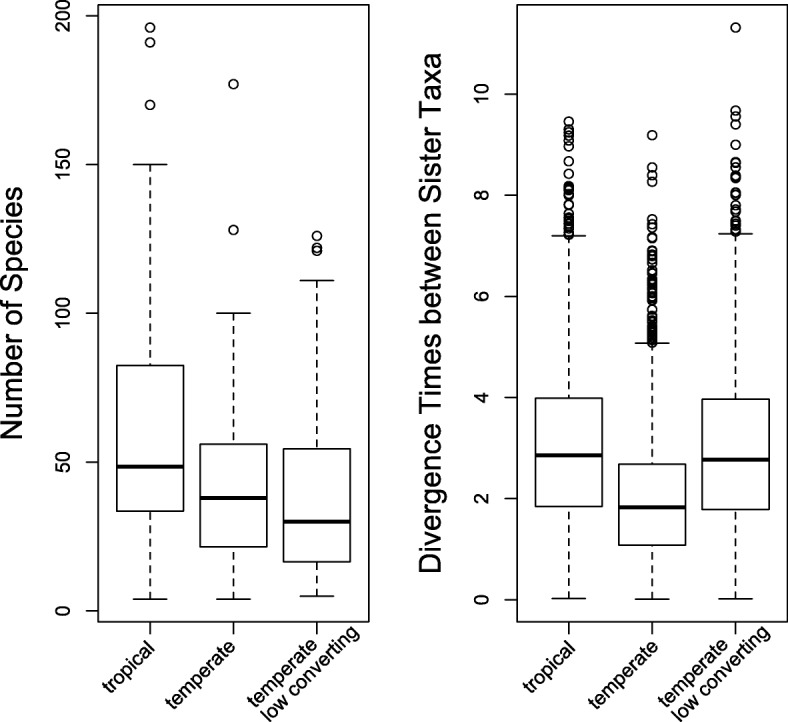
Bird latitudinal gradient results - despite differences in the underlying model for generating diversity, similar levels of species richness and gradient patterns were achieved. Left: species richness after 6 million years of protracted speciation for the tropical regions, temperate scenario 1 (high population splitting and conversion), and temperate scenario 2 (higher splitting and low conversion). Right: Mean cophenetic distances between sister species (i.e., sister species divergence time) for the same simulations

The simulation results not only show that microevolution level processes can result in a higher speciation rate in the temperate regions while maintaining the high species richness in the tropics, but also stress that different sets of microevolutionary parameters can generate similar gradient patterns (Fig. 2). For the first simulation using rate estimates derived from Weir and Schluter (2007), the average species richness for the temperate regions is 43.01 ± 2.72 (SE), as compared to 60.81 ± 4.12 for the tropical regions. The species richness is significantly higher in the tropical regions (*t* = 3.6085, *d.f.* = 171.45, and *P* < 0.001). The average sister species divergence time is 2.061 ± 0.14 for temperate regions and 3.027 ± 0.16 for tropical regions; the difference is also significant (*t* = 19.086, *d.f.* = 3312.6, and *P* < 0.001).

Under the second scenario, where a noticeably lower population conversion rate and a higher splitting rate were applied to the temperate regions, approximately the same number of species (38.62 ± 2.86) were generated as the first scenario. However, the differences can be shown via the mean sister species divergence time, where values from the second scenario (2.998 ± 0.17) were much higher (*t* = 15.99, d.f. = 2493.2, and *P* < 0.001; Fig. 2), indicating slower species conversion.

### Estimating speciation and extinction rates from protracted genealogies

**Fig. 3:**
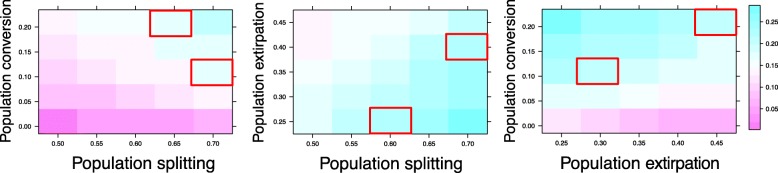
Birth-death estimation of speciation rates (color blocks) of phylogenies generated from 125 combination of protracted speciation parameters (rates of population splitting, population conversion, and population extirpation). Red squares represent examples of similar estimated speciation rates based on phylogenies generated from different protracted speciation parameters. Their different placement across parameter space shows that very different mechanism (e.g., different combinations of population splitting, conversion, and extinction rates) may underlie a single “speciation rate”, as measured under traditional macroevolutionary birth-death models

The estimated speciation rates for the 125 protracted parameter combinations range from 0.02–0.27. Speciation rates are the highest when rates of population splitting and conversion are high and the rate of population extirpation is low, whereas speciation rates are the lowest when the population extirpation rate is high and the other two rates are low (Fig. 3). However, very similar speciation rates can be estimated under different processes of divergence. For example, under the same population conversion rate (0.11), estimated speciation rate from a low population splitting (0.6), low extirpation (0.25) combination is the same as that of a high population splitting (0.7) and high extirpation (0.4) combination (Fig. 3, middle plot red squares). Similar patterns can be seen throughout the parameter space as similar colored blocks (i.e., speciation rates) are distributed widely, as with estimated extinction rates (see Additional file 1: Figure S1 and Table S1 for all estimated speciation and extinction rates).

## Discussion

### Empirical implications

Our results demonstrate how failing to account for the microevolutionary dynamics can impinge on our ability to understand the underlying cause of species diversity patterns, as demonstrated here through the context of latitudinal diversity gradients. Specifically, speciation and extinction events can be influenced by distinct ecological and biogeographic factors that ultimately affect the resulting speciation and extinction rates. In addition, different underlying dynamics can result in the same estimated speciation or extinction rates based on current macroevolutionary models. For example, as we show by reference to latitudinal gradients, a high speciation rate in the temperate zones can be driven by moderately high population splitting and conversion rates; however, the same species richness can be achieved by a combination of very high population splitting and low conversion rates. The former scenario indicates that species at higher latitudes may experience chronic divergent selection and often yield sister species pairs; the latter suggests frequent population fragmentation but low completion of speciation (also reflected in the divergence time between sister species). Both scenarios are probable and possibly co-occur given the harsher environments at higher latitudes [30], but cannot be distinguished based on speciation rates or species richness alone. Therefore, without examining diversification controls below the species level, the major underlying ecological mechanisms may never be fully recovered.

The implications of protracted speciation processes extend beyond the formation of latitudinal gradient. This framework might be essential for analyzing other significant macroevolutionary patterns; for instance, a striking species richness contrast exists between marine and terrestrial habitats - the ocean covers more than 70% of the earth surface but only harbors ~ 15% of the macroscopic species [43]. Study of amniote macroevolution have revealed that extant marine lineages often show higher rates of speciation compared to their terrestrial relatives, and their low species richness is more likely caused by older marine invasions’ inability to persist long term [44]. It is hypothesized that species-rich coastal habitats may exhibit higher environmental instability, resulting in high turnover in marine lineages, and this might be the major driver of the marine-terrestrial biodiversity gradient [44]. These speculated ecological mechanisms cannot be further disentangled unless we start to track within-species lineage diversifications. Neglecting protracted, multi-stage processes of diversification may hinder our ability to fully understand a great number of important ecological phenomena, such as biodiversity hotspots [45, 46] and major radiations [7, 47–49].

In addition, the protracted speciation framework enables us to discuss complex biological processes with clarity. For example, rates of reproductive isolation formation are not correlated with speciation rates in birds and *Drosophila* [50], even though it was often assumed that species which evolve reproductive isolations quickly should have higher speciation rates. This intuitive assumption would not be made if we see speciation as a combination of population splitting and conversion. Prezygotic isolation in the form of spatial separation is mostly driven by the population splitting process, it does not imply conversion into “good” species or even population persistence. Similarly, the formation of postzygotic isolation and other types prezygotic isolation (e.g. behavioral) is a sign of population conversion, and does not give information on the population splitting rates. Therefore, evolution of reproductive isolation is also a compound process. Its relationship with speciation rate can be better understood if the microevolutionary processes are considered.

### Establishing micro- and macroevolutionary links

Methodologies for incorporating microevolution into macroevolutionary analyses are still relatively limited, but promising developments have been seen in recent years. A maximum likelihood formula for protracted speciation has been developed [51] and applied to theoretical studies [32, 39]. Not all model parameters can be reliably estimated from phylogenetic data but the duration of speciation could be obtained without much bias [39]. The branching patterns in a phylogeny may also preserve some signatures of protracted speciation [36]. Estimating microevolutionary parameters based on phylogenies without comprehensive population level data are still challenging, but it is likely that future modeling development will start to accommodate incomplete population sampling. Incorporating microevolutionary modeling has been shown to improve predictions of the neutral theory of biodiversity [34]; generate alternative explanations for density dependent evolution [35], and illuminate trait macroevolution [52].

Empirical studies that examine the interaction between micro- and macroevolutionary processes are relatively rare (but see [50, 53–55]). Most works focus on small numbers of species or tend to use species richness data instead of phylogenetic information [45, 56]. Some empirical steps can be taken to gain protracted speciation parameters. Firstly, even though it is unrealistic to sample all existing within-species lineages when conducting large-scale phylogenetic studies, it would be beneficial to maintain some level of population sampling and include incipient species. The shape of such genealogies will give us information about population splitting rate [39]. Furthermore, even if populations can not be sampled, one can obtain population numbers and ages of well-studied species based on georeferencing and museum data. Once current population numbers and their ages are known, population splitting rate can be calculated assuming the splitting is a Poisson process [57]. Similar approaches can be used to gain population extirpation rate, but it requires population data to be collected consistently through time to detect extirpation, which may apply to some taxonomical groups, especially threatened or economically important species. As for population conversion rate, average sister species age [28] can be used as a proxy, although it is an underestimation of the conversion rate because what we identify today as sister species does not account for the complexities of extinction. Another possibility is to estimate the evolutionary rates of certain types of reproductive isolation among lineages [50]. This would require assessing mating behaviors and/or hybrid fertilities, which is possible in some systems. Overall, we need to be creative and combine diverse tools to link micro- and macroevolutionary research.

Lastly, given that the same birth-death model parameters can be associated with a diverse array of microevolutionary processes, one needs to be cautious when interpreting the biological meanings of macroevolutionary rates. Numerous studies have used macroevolutionary models to measure exceptional diversification rates or rate shifts in phylogenies (e.g., [11, 58–62]) and provided invaluable knowledge about the study systems. However, by coupling macroevolutionary rates with important ecological factors, biological traits, or geological events, conclusions were sometimes made to suggest these factors “promote” or “drive” the observed patterns. We would like to stress that given the compound nature of speciation and extinction rates, current methodologies may not have the power to resolve the mechanistic cause of certain macroevolutionary trends. It is important to ensure that the interpretation of the model is not divorced from what the model actually does. Strong correlations between ecological factors and macroevolutionary rates warrant further investigation of the underlying process, and microevolutionary dynamics is a crucial component that needs to be incorporated.

It should be noted that this study does not mean to undermine the importance of macroevolutionary research. It is crucial to understand macroevolutionary patterns and how dynamics of diversification rates are associated with biotic and abiotic factors. Distinct macroevolutionary patterns promote the development of new hypotheses and better investigation of lower-level biological processes. Just as speciation rates are controlled by population splitting and conversion, those population level processes are influenced by other factors, such as organisms’ behaviors and their genetic backgrounds. We would like to argue that the more we integrate processes at different scales, the better we can understand the biological system.

## Conclusion

Our analyses demonstrate that distinct microevolutionary scenarios can generate very similar and realistic biodiversity patterns (e.g., latitudinal diversity gradient). We also showed that current macroevolutionary models may not be able to distinguish these different scenarios. Therefore, inferring causal relationships between ecological factors and macroevolutioanry rates or patterns needs to be accompanied by rigorous assessments. Future studies that incorporate microevolutionary processes into current modeling approaches are in need.

## Additional file

Additional file 1: **Table S1.** All parameters used in simulating the protracted genealogies and all estimated speciation and extinction rates of these genealogies based on traditional birth-death models. **Figure S1.** Estimated extinction rates (color blocks) based on phylogenies generated from 125 combination of protracted speciation parameters (rates of population splitting, population conversion, and population extirpation). Note that this figure is different from Fig. 3, which reports on speciation rates. Red squares represent examples of similar estimated extinction rates based on phylogenies generated from different protracted speciation parameters. Their different placement across parameter space shows that very different mechanism (e.g., different combinations of population splitting, conversion, and extinction rates) may underlie a single “extinction rate”, as measured under traditional macroevolutionary birth-death models (ZIP 155 kb)
