# Supplementary material for: Microevolutionary processes impact macroevolutionary patterns
Source: BMC Evol Biol. 2018 Aug 10;18:123. doi: 10.1186/s12862-018-1236-8 (PMC6086068; doi:10.1186/s12862-018-1236-8)
Supplement: Supplementary file 1 — Table S1. All parameters used in simulating the protracted genealogies and all estimated speciation and extinction rates of these genealogies based on traditional birth-death models. Figure S1. Estimated extinction rates (color blocks) based on phylogenies generated from 125 combination of protracted speciation parameters (rates of population splitting, population conversion, and population extirpation). Note that this figure is different from Fig. 3, which reports on speciation rates. Red squares represent examples of similar estimated extinction rates based on phylogenies generated from different protracted speciation parameters. Their different placement across parameter space shows that very different mechanism (e.g., different combinations of population splitting, conversion, and extinction rates) may underlie a single “extinction rate”, as measured under traditional macroevolutionary birth-death models (ZIP 155 kb) [file 12862_2018_1236_MOESM1_ESM.zip › Supplementary_Figure _1.pdf]

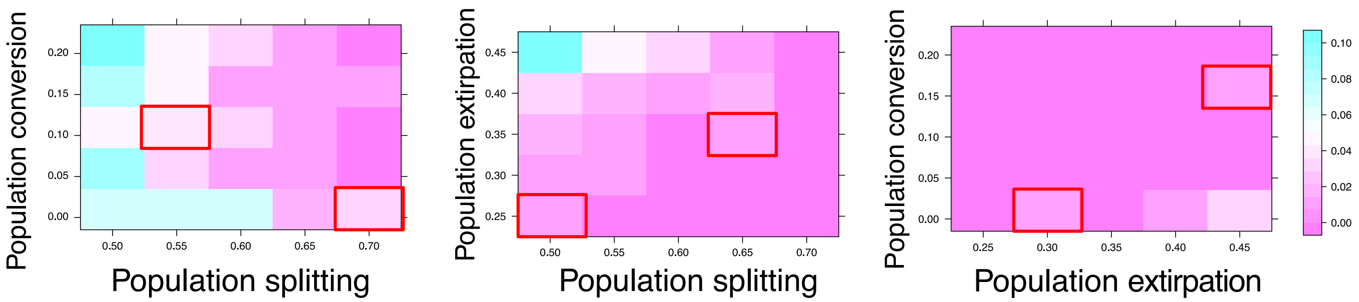

Supplementary Figure 1. Birth-death estimation of extinction rates (color blocks) of phylogenies generated from 125 combination of protracted speciation parameters (rates of population splitting, population conversion, and population extirpation). Red squares represent examples of similar estimated extinction rates based on phylogenies generated from different protracted speciation parameters. Their different placement across parameter space shows that very different mechanism (*e.g.,* different combinations of population splitting, conversion, and extinction rates) may underlie a single "extinction rate", as measured under traditional macroevolutionary birth-death models.
